# A novel biomarker of interleukin 6 activity and clinical and cognitive outcomes in depression

**DOI:** 10.1016/j.psyneuen.2024.107008

**Published:** 2024-02-29

**Authors:** Éimear M. Foley, Chloe Slaney, Nicholas A. Donnelly, Muzaffer Kaser, Louise Ziegler, Golam M. Khandaker

**Affiliations:** a MRC Integrative Epidemiology Unit, Population Health Sciences, Bristol Medical School, University of Bristol, Bristol, UK; b Centre for Academic Mental Health, Population Health Sciences, Bristol Medical School, University of Bristol, Bristol, UK; c Avon and Wiltshire Mental Health Partnership NHS Trust, Bristol, UK; d Cambridgeshire and Peterborough NHS Foundation Trust, Cambridge, UK; e Department of Psychiatry, University of Cambridge, Cambridge, UK; f Department of Clinical Sciences, Danderyd Hospital, Karolinska Institutet, Stockholm, Sweden; g NIHR Bristol Biomedical Research Centre, Bristol, UK

**Keywords:** Interleukin-6, Interleukin-6 Activity, Interleukin-6 Signalling, Inflammation, Depression, Cognition

## Abstract

**Background:**

Inflammatory cytokines like interleukin-6 (IL-6) are implicated in depression, but most studies have hitherto focused on circulating levels of IL-6 rather than its activity. IL-6 trans-signalling is thought to be responsible for most of the pathogenic effects of IL-6 and is implicated in autoimmune diseases like rheumatoid arthritis. We tested the association between a multi-protein-derived measure of IL-6 trans-signalling and clinical and cognitive outcomes in patients with depression. We hypothesised that this novel measure of IL-6 activity/bioavailability would be associated with clinical and cognitive measures previously reported to be associated with inflammation in depression.

**Methods:**

Using data from 86 patients with International Classification of Diseases-10 diagnosis of depression, we calculated a ratio score representing IL-6 activity/bioavailability using serum IL-6, soluble IL-6 receptor (sIL-6R), and soluble glycoprotein 130 levels. We tested the relationship of this novel biomarker with 12 cytokines using correlation analyses and with cognitive and clinical measures using multivariable linear regression, following z-transformation of all immune exposures.

**Results:**

The novel measure of IL-6 activity/bioavailability was correlated with IL-6 (r=0.42, P=0.03), C-reactive protein (CRP) (r=0.42, P=0.03), sIL-6R (r=0.91, P<0.01), and tumour necrosis factor alpha (r=0.43, P=0.03). The IL-6 activity/bioavailability measure was associated with higher somatic symptoms of depression (β=1.09; 95% CI 0.30, 1.88; *P*_*FDR*_=0.01), fatigue (β=4.34; 95% CI 1.26, 7.42; *P*_*FDR*_=0.03), depression severity (β=3.06; 95% CI 0.71, 5.40; *P*=0.02), poorer quality of life (β=−0.07; 95% CI −0.13, −0.01; *P*_*FDR*_=0.045), and decreased psychomotor speed (β=−5.46; 95% CI −9.09, −1.84; *P*_*FDR*_=0.01),. There was little evidence of associations with reaction time, anhedonia, anxiety, emotional perception and recall, executive function, and sustained attention (Ps>0.05). The effect estimates for the associations of the novel measure with depression outcomes were comparable to those for individual immune proteins (i.e., IL-6, CRP, sIL-6R).

**Conclusion:**

A novel multi-protein-derived measure of IL-6 activity/bioavailability shows robust associations with various inflammation-related clinical and cognitive outcomes in depression and performs well in comparison to single inflammatory proteins. We need replication of these findings in other samples, experiments for mechanistic validity of this novel biomarker, and clinical studies to assess its usefulness as a marker of illness risk and prognosis.

## 1 Introduction

Inflammation is implicated in the pathogenesis of major depressive disorder (MDD) and inflammatory cytokines are thought to play a central role. Interleukin-6 (IL-6), a pleiotropic proinflammatory cytokine, appears to be one of the most credible mechanistic candidates. Meta-analytic evidence confirms elevation in peripheral levels of IL-6 and acute-phase proteins, like C-reactive protein (CRP), in patients with depression compared to controls (Goldsmith et al., 2016; Köhler et al., 2017; Osimo et al., 2020). A large population-based prospective cohort study shows that childhood IL-6 levels are associated with depression risk in early-adulthood in a dose-response fashion (Khandaker et al., 2014). Mendelian Randomization (MR) analyses suggest that IL-6 may play a causal role in depression and somatic symptoms like fatigue (Khandaker et al., 2020; Ye et al., 2021; Kappelmann et al., 2021). Soluble IL-6 receptor (sIL-6R) has also been implicated in depression using MR analysis (Kelly et al., 2021). Meta-analyses of anti-cytokine treatments, including anti-IL-6/IL-6R medication, show a reduction of depressive symptoms in individuals with chronic inflammatory disease, independent of improvements to their physical illness (Kappelmann et al., 2018; Wittenberg et al., 2020). This evidence implicates inflammation in the aetiology of depression, suggesting that IL-6 could be a mechagnostic (mechanism-related) and theragnostic (treatment-response related) marker of depression.

While current evidence strongly suggests a role of IL-6 in depression, research to date has largely focused on circulating levels of IL-6 and other immune proteins rather than their activity, bioavailability, or specific signalling pathways. These measures are the products of dynamic interaction between multiple proteins and could be closer to the biological function/action of these proteins. Focusing on activity/bioavailability of IL-6 could help us better understand how IL-6 contributes to depression and may aid the development of strategies for precise therapeutic targeting of the IL-6 pathway in depression.

**Fig. 1:**
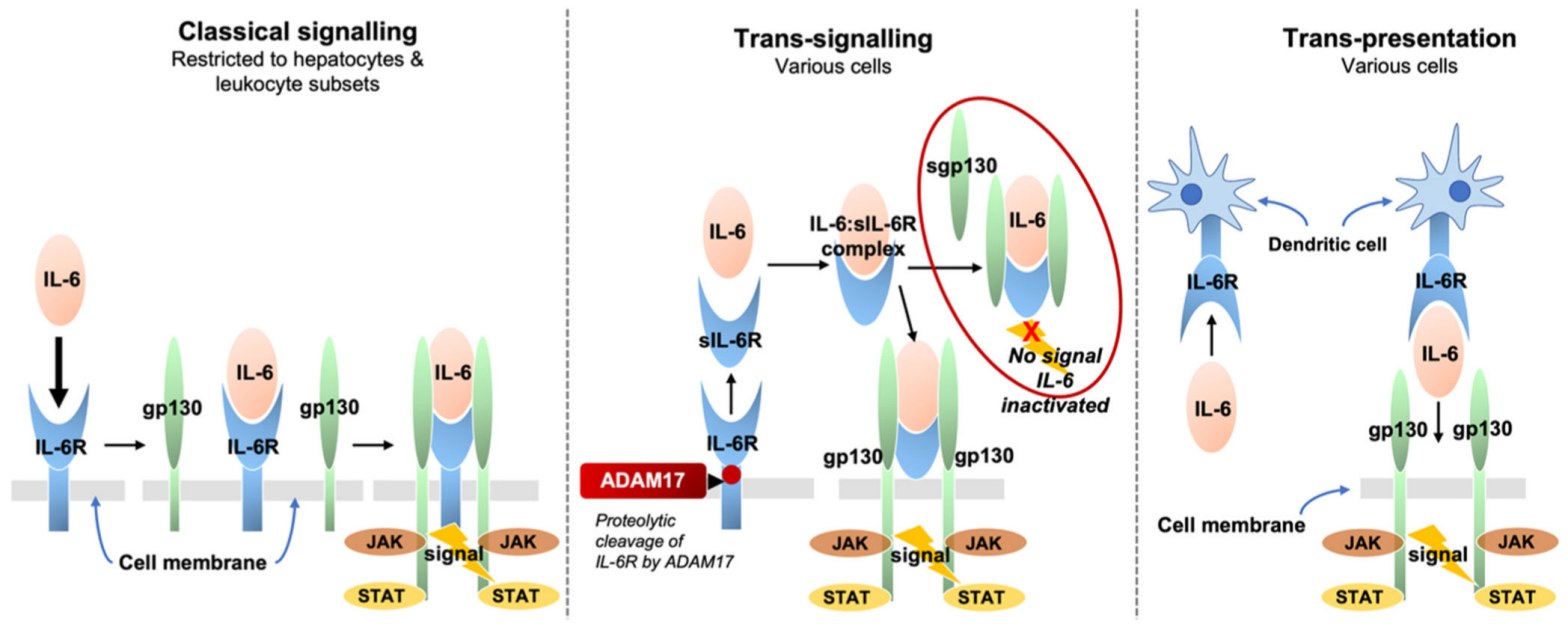
Mechanisms of interleukin-6 (IL-6) signalling. IL-6 signalling occurs via three routes: 1) Classical signalling is restricted to hepatocytes and leukocyte subsets that express gp130 via membrane-bound IL-6 receptor (IL-6R); 2) Trans-signalling can potentially occur in all cells via soluble IL-6R (sIL-6R); 3) Trans-presentation occurs in cells expressing gp130 via membrane-bound IL-6R on dendritic cells.

IL-6 exerts its biological effect through three signalling pathways (classical signalling, trans-signalling, and trans-presentation), which are products of the interaction between IL-6, IL-6R (or its soluble form sIL-6R), and glycoprotein 130 (gp130; or its soluble form sgp130) (see Fig. 1) (Heink et al., 2017; Schaper and Rose-John, 2015). IL-6 trans--signalling is thought to be responsible for most of the pathogenetic effects of IL-6 and is implicated in autoimmune diseases like rheumatoid arthritis and irritable bowel disease (Jarlborg and Gabay, 2022; Schreiber et al., 2021). In IL-6 trans-signalling, the relative abundance of sgp130 over IL-6:sIL-6R complex determines the availability of the IL-6: sIL-6R complex free to trigger trans-signalling. This is because sgp130 can bind with IL-6:sIL-6R complex to form a ternary complex (IL-6: sIL-6R:sgp130) leading to its neutralisation.

Based on this insight into IL-6 signalling biology, Ziegler and colleagues used circulating IL-6, sIL-6R, and sgp130 levels to develop a measure that reflects IL-6 activity/bioavailability or trans-signalling strength (see Methods Section 2.3.2) (Ziegler et al., 2019). Using Swedish population cohort data, they showed that this novel measure of IL-6 activity is associated with increased risk of future cardiovascular events (Miri et al., 2021; Ziegler et al., 2019) and ischemic stroke (Ziegler et al., 2021). Elsewhere, this ratio has been associated with coronary artery disease in postmenopausal women (Zhou et al., 2020). While this measure could be useful for studying the biological functions of IL-6, possibly trans-signalling in particular, it remains unclear whether this measure is associated with the clinical and cognitive features of depression that have previously been linked with inflammation.

The aim of the current study was to examine: 1) the relationship between a novel multi-protein derived measure of IL-6 activity/bioavailability and other relevant immune markers; 2) the relationship between IL-6 activity/bioavailability and inflammation-related clinical and cognitive outcomes in depression; and 3) how associations for this novel biomarker with depression outcomes compare to those for relevant single immune proteins, such as IL-6, CRP, and sIL-6R.

## 2 Materials and methods

### 2.1 Participants and study design

This study involved individuals with an International Classification of Diseases 10th Revision (ICD-10) diagnosis of depression (code F32) recruited as part of a randomised clinical trial, The Insight Study. The full study protocol has previously been published (Khandaker et al., 2018). This trial aimed to recruit roughly equal number of participants with low (<3 mg/L) and high (≥3 mg/L) high sensitivity (hs)-CRP levels. This cut-off was chosen based on the American Heart Association and Center for Disease Control and Prevention recommendations, which defined CRP levels of >3 mg/L as high (Pearson et al., 2003). The high CRP group was enriched for somatic symptoms by design, and this group was later randomised to receive an anti-inflammatory drug or placebo. The analyses presented here comprise data from all Insight Study participants at baseline regardless of CRP levels or subsequent randomisation, as the study was also designed to allow observational research into inflammation and depression (Khandaker et al., 2018). Recruitment strategy for both groups was identical. All patients were recruited through United Kingdom National Health Service Mental Health Trusts, primary care general practice surgeries, and self-referrals from the East Anglia region of England between October 2018 and June 2022. The high CRP group were tested for acute infection using blood tests (i.e., white blood cell count, antibody tests for TB, HIV, Hepatitis B, and Hepatitis C) and a chest X-ray, and those with evidence of infection were excluded. All participants were taking an antidepressant at an adequate dose (according to the British National Formulary) for at least four weeks prior to recruitment and continued this medication throughout their study participation. See Supplementary Materials for full study inclusion and exclusion criteria.

Demographic and medical history information was recorded, and self-administered psychiatric evaluations were completed during the baseline study visit. Participants also provided blood samples during this same testing session. Informed consent was obtained for all participants prior to testing. The study was approved by the South Central - Oxford B Research Ethics Committee (Reference: 18/SC/0118).

### 2.2 Diagnosis of depression

All participants met the ICD-10 criteria for depressive episode at the time of participation as assessed by the Clinical Interview Schedule - Revised (CIS-R). The CIS-R is a widely used, standardised tool for measuring common mental health disorders in research settings (Lewis, 1994). The CIS-R is a fully structured assessment suitable for trained interviewers and does not require any expert knowledge on the part of the interviewers. As such, it can also be administered using personal computers on which the subjects self-complete the questionnaire, supported by trained research staff. The CIS-R elicits responses to 14 areas of symptoms i.e., fatigue, appetite, sleep problems, concentration difficulties, irritability, depression, depressive ideas, anxiety, worry, panic, phobia, compulsive behaviours, obsessive thoughts, and somatic symptoms. It can be used to generate diagnostic categories according to ICD-10 criteria, including diagnosis of depression.

### 2.3 Exposure measurement

#### 2.3.1 Inflammatory proteins

Blood samples were collected from non-fasting participants, which were analysed at the Core Biochemical Assay Laboratory at the Addenbrooke’s Hospital, Cambridge, United Kingdom. Laboratory staff were blind to psychiatric data and measurements were performed according to manufacturers’ protocols. All blood samples were transported directly to laboratory on the day of collection. Please see Supplementary Table 1 for the upper and lower detection limits for each inflammatory protein. Immune markers with values below the lower limit of detection (e.g., IL-6 <0.5 pg/mL) were assigned the corresponding lower limit value (e.g., IL-6 = 0.5 pg/mL). No proteins had values above their corresponding upper limit of detection.

Upon collection, blood samples were promptly centrifuged at 1600 g for 15 minutes at room temperature. Serum was transferred into pre-labelled 500 μl aliquots and frozen at –70°C. Immunoassay panels designed prior to participant recruitment were run on trial blood samples collected at baseline. Serum hs-CRP levels were obtained using an automated colorimetric immunoassay on the Siemens Dimension EXL analyser. Serum interferon (IFN)-γ, IL-1β, IL-2, IL-4, IL-6, IL-8, IL-10, IL-12p70, IL-13, and tumour necrosis factor (TNF)-α were measured using the MesoScale Discovery (MSD) 10-plex Human Proinflammatory Panel. SIL-6R was measured using a single MSD R-Plex assay and an R&D Quantikine ELISA was performed to measure sgp130. Serum sIL-6R and sgp130 samples were diluted to 1:50 and 1:1000, respectively. Sgp130 was assayed separately for high and low CRP groups but there was no evidence of large batch effect. Distribution, mean (SD), and median (IQR) of sgp130 were comparable between two sets of analyses (Supplementary Table 2; Supplementary Figure 1).

#### 2.3.2 Calculation of a novel marker of IL-6 activity/bioavailability

Serum IL-6 concentrations were converted from pg/mL to ng/mL by dividing by 1000. Next, molar concentrations (i.e., mol/L) for IL-6, sIL-6R, and sgp130 were calculated by dividing their serum concentrations (ng/mL) by their molecular weights in kilo Dalton (i.e., IL-6 by 23.7, sIL-6R by 50, sgp130 by 100). Binary (IL-6:sIL-6R) and ternary (IL-6:sIL-6R: sgp130) complexes were calculated using the formulas reported previously (Garbers et al., 2011; Müller-Newen et al., 1998).

**Binary Complex [IL6 : sIL6R]**

$$\begin{array}{l} 0.5[\text{sIL}6\text{R}]\text{i}+0.5[\text{IL}6]\text{i}+0.5{\text{K}}_{\text{D}1}\\ -0.5{\left([\text{sIL}6\text{R}]{\text{i}}^{2}+[\text{IL}6]{\text{i}}^{2}+2[\text{IL}6]\text{i}{\text{K}}_{\text{D}1}+{\text{K}}_{\text{D}1}^{2}\right)}^{0.5} \end{array}$$

**Ternary Complex [IL6 : sIL6R : sgp130]**

$$\begin{array}{l} 0.5[\text{sgp}130]\text{i}+0.5[\text{IL}6:\text{sIL}6\text{R}]\text{i}+0.5{\text{K}}_{\text{D}2}\\ -0.5{\left([\text{sgp}130]{\text{i}}^{2}+[\text{IL}6:\text{sIL}6\text{R}]{\text{i}}^{2}+2[\text{IL}6:\text{sIL}6\text{R}]\text{i}{\text{K}}_{\text{D}2}+{\text{K}}_{\text{D}2}^{2}\right)}^{0.5} \end{array}$$

Molar concentrations of serum IL-6, sIL-6R, and sgp130 are represented by [IL6]i, [sIL6R]i, and [sgp130]i, respectively. Dissociation constants (K_D_) are a measure of binding affinity and are represented by K_D1_ for the binary complex and K_D2_ for the ternary complex. Binding affinity is the strength of the interaction between two or more molecules. Therefore, K_D1_ represents the strength of interaction between IL-6 and sIL-6R while forming the binary complex, and K_D2_ represents the strength of interaction between the binary complex and sgp130 while forming the ternary complex. As the binary complex is neutralised (and IL-6 signalling is prevented) when it binds with sgp130 to form the ternary complex, in this context increased binding affinity for the ternary complex (i.e., K_D2_) corresponds with reduced IL-6 trans-signalling. Similarly, a lower binding affinity for the binary complex would also correspond with reduced IL-6 trans-signalling (because IL-6 trans-signalling occurs when the binary complex binds with gp130 in cell membrane). We have used previously reported values forK_D1_ and K_D2_, estimated as 0.5 and 0.05 nmol/L, respectively (Garbers et al., 2011). IL-6 activity/bioavailability was then calculated by dividing the binary complex by the ternary complex (i.e., IL-6:sIL-6R / IL-6:sIL-6R:sgp130) and used as the primary exposure in our analyses. We also carried out sensitivity analyses by substituting the values for K_D1_ and K_D2_ with values 10 times higher and 10 times lower than the original values (above) while calculating the ratio between the binary and ternary complexes (Ziegler et al., 2019). Total depression severity score was used as the outcome for the sensitivity analysis (see below).

### 2.4 Outcome measurement

All primary and secondary outcome measures are described (below) and tabulated (Supplementary Table 3).

#### 2.4.1 Clinical outcomes

Participants completed self-administered validated questionnaires for depression, fatigue, anhedonia, state and trait anxiety, and quality of life. Total scores for each test were calculated by summing individual item scores according to user manuals. For all questionnaires, higher scores represented greater symptom severity, except for quality of life (see below). We used Cronbach’s alpha to quantify internal consistency of clinical outcomes in the current study (Cronbach, 1951); Cronbach’s alpha of ≥0.90 was considered as excellent, 0.8–0.9 as good, 0.7–0.8 as acceptable, 0.6–0.7 as questionable, 0.5–0.6 as poor, and <0.5 as unacceptable.

##### 2.4.1.1 Depression severity and somatic symptoms

Depressive symptoms were assessed using the Beck’s Depression Inventory (BDI)-II (Beck et al., 1996). Each item on this 21-item questionnaire was coded on a 4-point scale ranging from 0 to 3 giving a total score of 0–63. Cronbach’s alpha for the BDI-II was 0.89, indicating high internal consistency in this sample. Elevated cytokine levels have been linked to higher depression severity in general (Köhler-Forsberg et al., 2017), and in particular with somatic symptoms of depression, like fatigue, and altered sleep or appetite (Milaneschi et al., 2021; Chu et al., 2019). Therefore, we additionally calculated a somatic symptom score with a total score of 0–21 by summing seven relevant BDI-II items (Foley et al., 2021), specifically: 4 = lack of pleasure, 15 = loss of energy, 16 = changes in sleeping pattern, 18 = changes in appetite, 19 = concentration difficulty, 20 = tiredness or fatigue, and 21 = loss of interest in sex. Cronbach’s alpha for the somatic symptom score was 0.69, indicating acceptable internal consistency in this sample. Somatic symptom score was used as the primary outcome for this study.

##### 2.4.1.2 Fatigue, anhedonia, and anxiety

Fatigue is a common symptom of depression and has previously been shown to be strongly associated with inflammation (Chu et al., 2019; Milaneschi et al., 2021). Therefore, fatigue was also included as a primary outcome in the current study. Fatigue was assessed using the 20-item Multidimensional Fatigue Inventory (MFI) (Smets et al., 1995). Item scores were coded on a 5-point scale ranging from 1 to 5 with a total score of 20–100 (Cronbach’s α = 0.91). Higher scores indicated greater fatigue severity.

In addition, the Snaith-Hamilton Pleasure Scale (SHAPS) (Snaith et al., 1995) was used to measure anhedonia. Items on this 14-item scale were coded as 0 = agree and 1 = disagree with a total score of 0–14 (Cronbach’s α = 0.82). The State-Trait Anxiety Inventory (STAI) (Speilberger et al., 1983) was also used to measure state (STAI-S) and trait (STAI-T) anxiety. Both questionnaires demonstrated good internal reliability with a Cronbach’s alpha of 0.92 and 0.88, respectively. Participants were presented with two 20-item questionnaires to assess these two forms of anxiety. Responses were recorded on a 4-point scale ranging from 1 to 4, giving a total score of 20–80 per questionnaire.

##### 2.4.1.3 Quality of life

Quality of life was assessed using the EuroQol five-dimension three-level version (EQ-5D-3 L) (EuroQol Research Foundation, 2018). The EQ-5D-3 L assesses five dimensions of quality of life, namely, mobility, self-care, usual activities, pain/discomfort, and anxiety/depression (Cronbach’s α = 0.64). Participants assigned each dimension a score ranging from 1 to 3, with higher scores indicating poorer quality of life. Combining these numbers in sequence resulted in a five-digit health state profile that represented the level of reported problems on each of the five dimensions. Health state profiles were converted into a single index value to reflect participants’ overall quality of life, scored from around 0 to maximum 1, with 1 representing perfect health.

#### 2.4.2 Cognitive outcomes

Cognitive tasks were selected to represent cognitive domains known to be affected in depression. All tests are well-established regarding reliability and sensitivity. Unless otherwise states, outcome variables from all tests were coded to ensure lower scores represented worse performance.

##### 2.4.2.1 Cold cognition

Inflammation-related depression is reported to be associated with cognitive dysfunction, particularly decreased psychomotor speed and reaction time (Kaser et al., 2022; Krogh et al., 2014). Therefore, we included these two domains as primary outcomes in our analyses. All other test outcomes were included as secondary outcomes. Psychomotor speed was assessed using a digit symbol coding test. In this task, participants were asked to copy symbols corresponding to numbers as quickly and as accurately as possible within 90 seconds. The outcome measure was number of correctly coded symbols. Reaction time was assessed using the Cambridge Neuropsychological Test Automated Battery (CANTAB) (Cambridge Cognition, 2019) Reaction Time Test five-choice mode. Upon the appearance of a target, participants were asked to react as quickly as possible by releasing a button at the bottom of the screen and selecting the circle in which a dot had appeared. The outcome measure was median reaction time.

We additionally assessed several other cognitive domains: visual associative learning and memory, executive function, and sustained attention. Visual associative learning and memory was assessed using the CANTAB Paired Associates Learning test. Objects were momentarily displayed in boxes shown on screen in a random order. Participants were then shown each object individually and asked to identify which box they were originally located in. The outcome measure was the total number of errors adjusted (i.e., total number of errors plus an adjustment for number of stages not reached). Executive function (i.e., spatial planning and working memory subdomains) was assessed using the CANTAB One Touch Stockings of Cambridge test. Participants were presented with three coloured balls stacked in two different patterns. They were asked to decipher the minimum number of moves required to match one pattern with another. The outcome measure was number of problems solved in first choice. Lastly, the CANTAB Rapid Visual Information Processing test was used to assess sustained attention. Participants are asked to detect target sequences of digits (e.g., 2–4–6, 4–7–9) that appeared among other rapidly presented (100 digits/minute) pseudo-random digits (ranging from 2 to 9). The outcome measure for this task was median response latency.

##### 2.4.2.2 Hot cognition

Two tests assessed affective bias: The Emotional Categorisation and Recall Task (ECAT) and the CANTAB Emotion Bias Task (EBT). The ECAT was used to assess affective bias and comprised of two stages: categorisation and recall. In the categorisation stage, participants are presented with 60 words of changing valence, 30 positive (e.g., cheerful) and 30 negative (e.g., hostile), and asked to categorise these words as likeable or dislikeable personality traits. The outcome measures for this stage of the task were reaction time for positive and negative words. After a delay of 15 minutes, participants were then asked to recall as many of the 60 words as possible. The outcome measures for this recall stage were total positive and total negative words recalled. The EBT (happy-to-sad variant) was used to assess perceptual bias in facial emotion perception. Participants are briefly shown faces (150 ms) morphed between two emotions (happy and sad) of varied intensities and are asked to identify the emotion displayed using a two-alternative forced choice. The outcome measure was bias point, i.e., the proportion of trials selected as happy compared to sad, adjusted to a scale of 0–15. Higher scores reflect a positive bias.

### 2.5 Assessment of covariates

We used self-report questionnaires to measure age, sex, number of depressive episodes, current antidepressant type (selective serotonin reuptake inhibitor vs other), and treatment duration (weeks). When asked about their total number of depressive episodes, 17 participants responded “Don’t know”. However, participants provided information on depression treatment history including current and previous anti-depressant medication name, class, dose, and duration, and other relevant treatments. We used this information to estimate the probable number of depressive episodes for these participants. Body mass index (BMI) was calculated from height and weight measurements taken by staff during the study visit and included as a covariate in our clinical analyses. Intellectual ability is an important and relevant confounder for inflammation and cognition-related outcomes. Therefore, estimated premorbid IQ was assessed using the National Adult Reading Test (NART) and was included as a covariate in our cognitive analyses. The NART is comprised of 50 words with irregular spellings in British English (e.g. “aisle”). Participants were asked for the pronunciation of these words and scores were then converted, based on manual equations, to predict IQ scores on the Weschler Adult Intelligence Scale (Bright et al., 2018).

### 2.6 Data analysis

Statistical analyses were performed in R version 4.3.0 (R Core Team, 2020). The sample comprised 86 participants, allowing for 80% statistical power to detect medium effect sizes of Cohen’s *d*=0.17 (alpha=0.05). Non-normally distributed variables were log_10_--transformed (positive skew) or cube-transformed (negative skew) before analysis.

#### 2.6.1 Correlation analyses

To test the relationship between the novel IL-6 activity/bioavailability marker and other relevant inflammatory protein measures, we performed correlation analyses and plotted our results in a correlation matrix.

#### 2.6.2 Multivariable linear regression

All inflammatory exposure variables were z-transformed to allow for comparison of scores. Primary analyses were performed using multi-variable linear regression to assess the association between the novel IL-6 activity/bioactivity measure and the aforementioned clinical and cognitive depression outcomes. Three regression models were run in each instance: Model 1 = unadjusted, Model 2 = adjusted for BMI (clinical analyses) or NART score (cognitive analyses), and Model 3 = additionally adjusted for age, sex, current anti-depressant type, treatment duration, and number of depressive episodes. We applied false discovery rate (FDR) corrections for multiple comparisons using the Benjamini-Hochberg method (Benjamini and Hochberg, 1995). P-values were corrected based on total number of exposures tested per outcome (i.e., four exposures, IL-6 activity/bioavailability, IL-6, CRP, and sIL-6R, per outcome).

Secondary analyses were then performed as above to allow for comparisons between the novel biomarker and relevant single immune proteins, IL-6, CRP and sIL-6R. Beta estimates and confidence intervals were then compared for each exposure per outcome and the results tabulated and plotted.

### 2.7 Results

#### 2.7.1 Sample characteristics

Sample characteristics are presented in Table 1. The sample comprised 86 patients with ICD-10 diagnosis of depression (mean age = 38.36±11.79; 72% female; mean BMI = 30.62±8.80). The mean (SD) and median (interquartile range) for each inflammatory protein assessed are presented (Supplementary Table 4). All analyses were completed using maximum data available, and thus, sample size for each analysis differs based on data availability. Total sample size for each analysis is reported. Multivariable linear regression results for each model are presented (Table 2; Supplementary Table 5).

#### 2.7.2 Novel multi-protein-derived biomarker of IL-6 activity/bioavailability

**Fig. 2:**
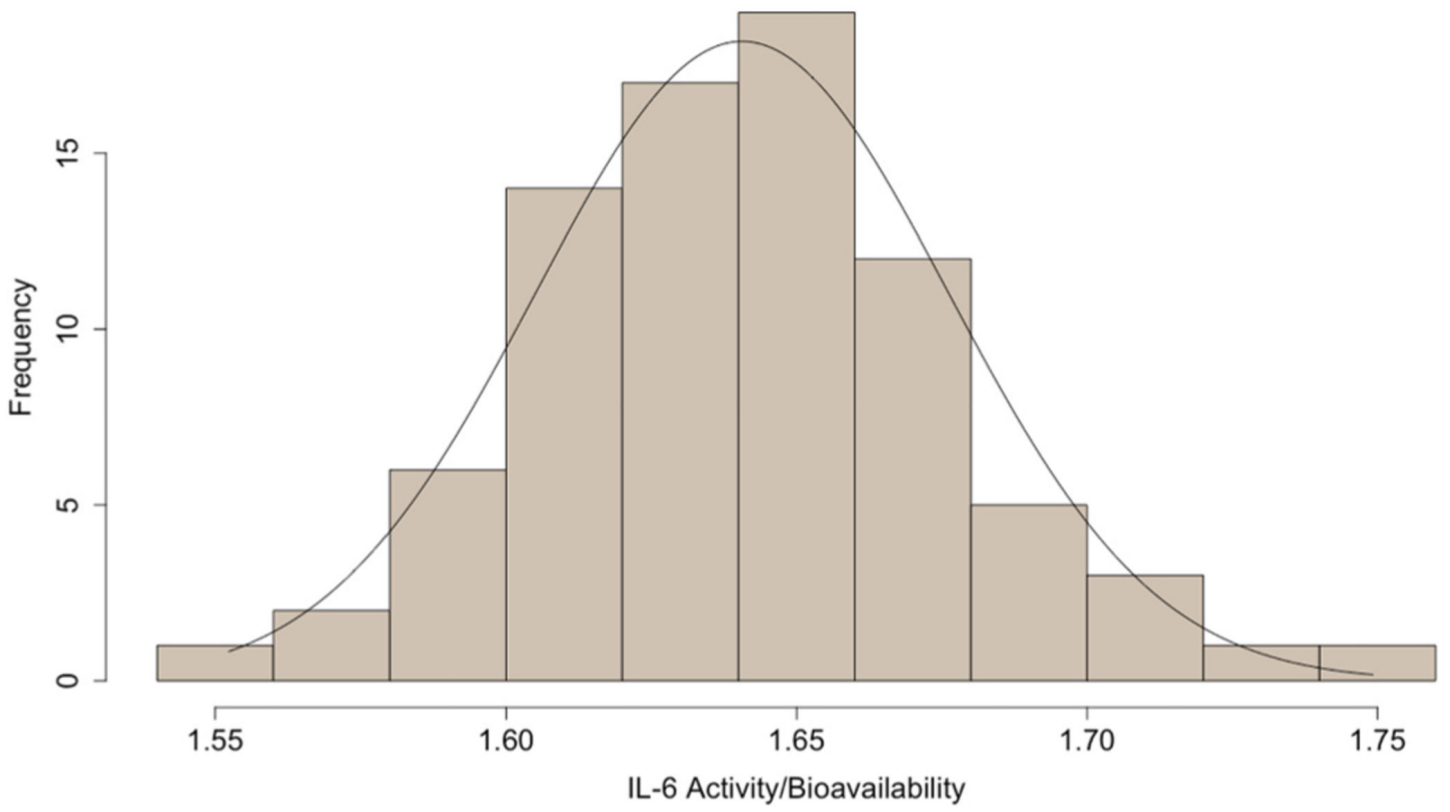
Distribution of novel biomarker of IL-6 activity/bioavailability in patients with depression. Note: This biomarker is a ratio score calculated using levels of interleukin-6 (IL-6), soluble IL-6 receptor (sIL-6R), and soluble gp130 (sgp130) and represents IL-6 activity/bioavailability (see Methods 2.3.2).

The novel biomarker of IL-6 activity/bioavailability was calculated using available data from 81 participants. The distribution for this variable was approximately normal (mean=1.64, SD=0.04, min=1.55, max=1.75) (Fig. 2). This multi-protein-derived measure was positively correlated with CRP (r=0.42, P=0.03), IL-6 (r=0.42, P=0.03), sIL-6R (r=0.91, P<0.01), and TNF-α (r=0.43, P=0.03), and negatively correlated with sgp130 (r=–0.31, P=0.07) (Supplementary Figure 2). Sensitivity analyses using binding affinities 10 times greater and lower than the value used for primary calculations showed no differences in the relationship of this measure with depression severity score (Supplementary Table 6 **and** Supplementary Figure 3).

#### 2.7.3 Association between IL-6 activity/bioavailability and clinical and cognitive outcomes

Analyses of primary outcomes showed an association between higher IL-6 activity/bioavailability and higher somatic symptoms (fully adjusted β or β_adj_=1.09; 95% CI 0.30, 1.88; *P*_*FDR*_=0.01), higher fatigue (β_adj_=4.34; 95% CI 1.26, 7.42; *P*_*FDR*_=0.03), and decreased psychomotor speed (β_adj_= −5.46; 95% CI −9.09, −1.84; *P*_*FDR*_=0.01) (Table 2). IL-6 activity/bioavailability was not strongly associated with reaction time (β_adj_=5.88; 95% CI −6.13, 17.89; *P*_*FDR*_=0.51).

Analyses of secondary outcomes showed an association between higher IL-6 activity/bioavailability and higher depression severity (β=3.06; 95% CI 0.71, 5.40; *P*_*FDR*_=0.02) and poorer quality of life (β=−0.07; 95% CI −0.13, −0.01; *P*_*FDR*_=0.045). The novel marker of IL-6 activity/bioavailability was found to be associated with poorer visual associative learning and memory in the unadjusted model, before correction for multiple testing (β_unadj_=−2.72; 95% CI −5.35, −0.10; *P*=0.04). However, upon the addition of covariates, confidence intervals widened and included the null. (Table 2). There was little evidence that IL-6 activity/bioavailability was associated with anhedonia, anxiety, emotional perception, reaction time (positive and negative words), and recall, executive function, and sustained attention (Supplementary Table 5).

#### 2.7.4 Association between individual inflammatory markers (IL-6, CRP, and sIL-6R) and clinical and cognitive outcomes

Higher IL-6 was associated with higher somatic symptoms (β_adj_=1.92; 95% CI 0.87, 2.97; *P*_*FDR*_=1.71e-03) and decreased psychomotor speed (β_adj_=−5.12; 95% CI −8.72, −1.53; *P*_*FDR*_=0.01), with weaker evidence for fatigue (β_unadj_=4.06; 95% CI −0.20, 8.32; *P*_*FDR*_=0.08) (Table 2). Higher CRP was associated with higher somatic symptoms (β_adj_=1.48; 95% CI 0.63, 2.33; *P*_*FDR*_=1.71e-03) and higher fatigue (β_adj_=3.80; 95% CI 0.53, 7.07; *P*_*FDR*_=0.047). Higher sIL-6R was associated with decreased psychomotor speed (β_adj_=−6.03; 95% CI −9.76, −2.30; *P*_*FDR*_=0.01). There was little evidence for associations of i) IL-6 with reaction time, ii) CRP with reaction time or psychomotor speed, and iii) sIL-6R with somatic symptoms, fatigue, or reaction time (Table 2).

Analyses of secondary outcomes showed associations between higher IL-6 and higher depression severity (β_adj_=4.80; 95% CI 1.62, 7.97; *P*_*FDR*_=0.01) and poorer quality of life (β_adj_=−0.12; 95% CI −0.21, −0.04; *P*_*FDR*_=0.01), with weaker evidence for an association with higher state anxiety (β_adj_=3.63; 95% CI 0.09, 7.18; *P*=0.04; *P*_*FDR*_=0.18) (Table 2; Supplementary Table 5). Higher CRP was associated with higher depression severity (β_adj_=3.33; 95% CI 0.80, 5.86; *P*_*FDR*_=0.02) and poorer quality of life (β_adj_=−0.09; 95% CI −0.16, −0.03; *P*_*FDR*_=0.01). Little evidence for an association was found for i) IL-6 with anhedonia, trait anxiety, associative learning and memory, emotional perception, reaction time (positive and negative words), and recall, executive function, or sustained attention, ii) CRP with anhedonia, anxiety, associative learning and memory, emotional perception, reaction time (positive and negative words), and recall, executive function, or sustained attention, and iii) sIL-6R with depression severity, quality of life, anhedonia, anxiety, associative learning and memory, emotional perception, reaction time (positive and negative words), and recall, executive function, or sustained attention (Table 2; Supplementary Table 5).

#### 2.7.5 Effect size comparison between novel IL-6 activity measure and individual inflammatory markers on clinical and cognitive outcomes

**Fig. 3:**
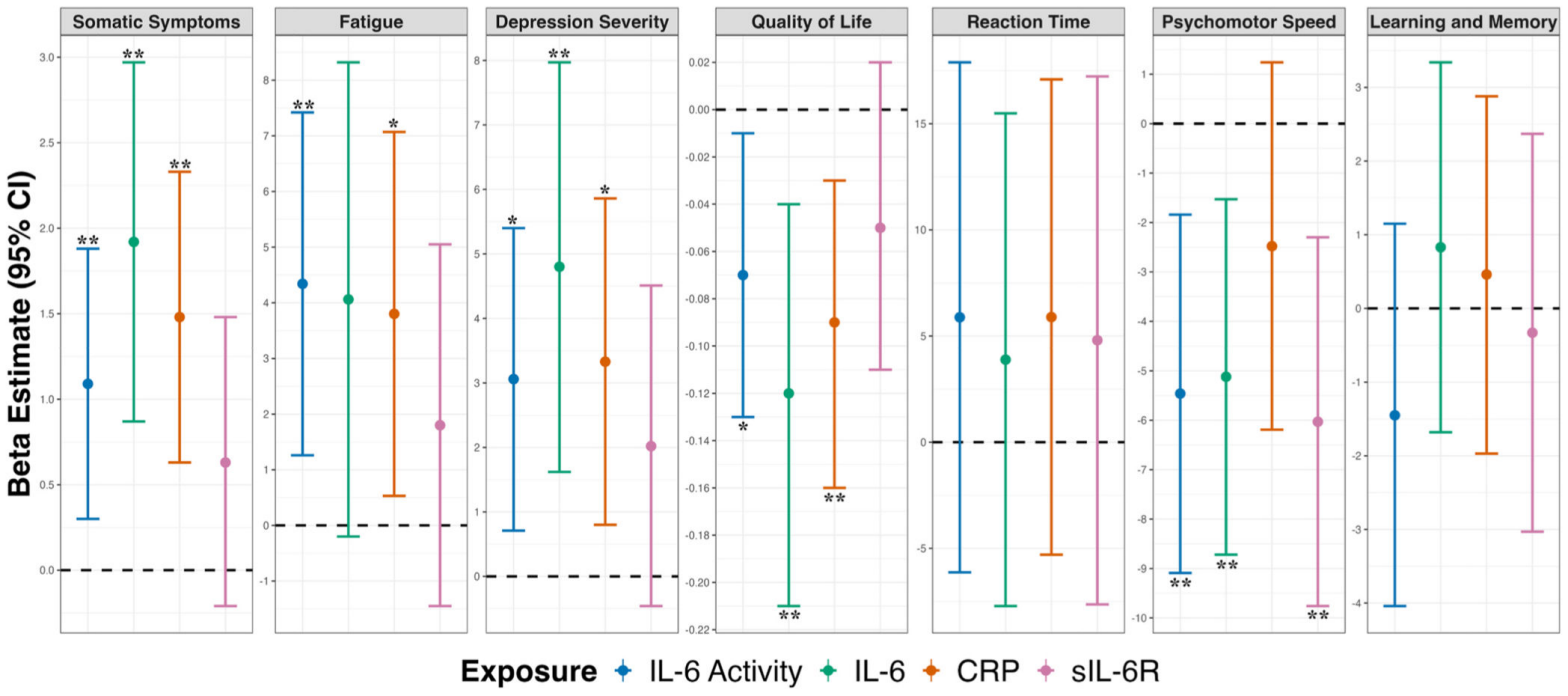
Comparison of the effects of novel IL-6 activity measure and other biomarkers on clinical and cognitive outcomes. *Note:* Adjusted for age, sex, current anti-depressant type, treatment duration, number of depressive episodes, and either BMI (clinical outcomes) or NART score (cognitive outcomes). All inflammatory exposures z-transformed. CRP = C-reactive protein; IL = interleukin; sIL-6R = soluble IL-6 receptor; * P<0.05 prior to FDR correction; ** P<0.01 prior to FDR correction.

The effect sizes and confidence intervals for somatic symptoms, fatigue, depression severity, and quality of life were comparable between IL-6 activity/bioavailability and CRP (Table 2**;** Fig. 3). Effect sizes for IL-6 were larger for select outcomes (i.e., somatic symptoms, depression severity, quality of life) than those for the other immune markers and confidence intervals were typically wider, crossing the null for fatigue. The effect sizes and confidence intervals for psychomotor speed were comparable between the IL-6 activity/bioavailability and IL-6 and sIL-6R, while these were smaller and wider, respectively, for CRP, crossing the null. IL-6 activity/bioavailability and, to a lesser extent, sIL-6R showed negative effects for learning and memory, while IL-6 and CRP were positive. However, confidence intervals overlapped and crossed the null for all markers. Effect sizes and confidence intervals were comparable across all four immune markers for reaction time and all other clinical and cognitive outcomes assessed (Table 2**;** Fig. 3**;** Supplementary Table 5).

#### 2.7.6 Results of sensitivity analyses

Sensitivity analyses were conducted to assess whether the ratio score was primarily driven by an individual inflammatory marker used to create the measure (i.e., IL-6, sIL-6R, sgp130). Three new ratio scores were generated, each with an individual protein increased threefold in value. The new ratio scores were then used as the exposure in our above-described unadjusted multivariable linear regression analyses with depression severity as the outcome. No single inflammatory marker was identified as the primary driver of the ratio score (Supplementary Table 7).

## 3 Discussion

We have derived a novel, multi-protein biomarker of IL-6 activity/bioavailability in a sample of patients with an ICD-10 diagnosis of depression. This biomarker shows robust associations with several clinical (i.e., somatic symptoms, fatigue, depression severity, quality of life) and cognitive (i.e., psychomotor speed) outcomes and performs well in comparison to single inflammatory proteins, like IL-6, CRP, and sIL-6R. The use of measures that reflect inflammatory marker activity could prove useful for understanding the underlying immune-related mechanisms for depression and identifying novel treatment targets.

The novel ratio measure used in this study was derived based on the knowledge of IL-6 signalling biology and represents IL-6 activity/bioavailability or strength of IL-6 trans-signalling. Previously, this ratio measure was shown to be associated with risk of future cardiovascular events, coronary artery disease in postmenopausal women, and ischemic stroke in a Swedish cohort (Miri et al., 2021; Zhou et al., 2020; Ziegler et al., 2021, 2019). Following the same methods, we have calculated this ratio measure in a sample of patients with depression. We have shown that this novel biomarker is correlated with several individual proteins, including CRP, TNF-α, IL-6, sIL-6R and spg130 – but it is important to note that the latter three proteins were used in the creation of this ratio score. Nonetheless, it is reassuring that the novel measure of IL-6 activity/bioavailability correlates with more established inflammatory markers like CRP. Studies of CRP and other individual inflammatory markers have been invaluable in establishing the link between inflammation and depression and have furthered our understanding of the role cytokines play in the development and persistence of depression. We have also shown associations between the ratio measure and several inflammation-related phenotypes in depression, such as fatigue, somatic symptoms, and depression severity. This novel biomarker integrates multiple proteins, their properties, and binding affinities and, therefore, could reflect a better representation of the biological function/action of IL-6. Using this measure in addition to individual proteins in future research may provide novel insights into the role inflammation plays in depression and related psychiatric disorders.

There are many valid ways to measure inflammatory status or immune system activity. We have previously highlighted the potential usefulness immune cells as biomarkers for depression (Foley et al., 2022), while others have reported neutrophil-lymphocyte ratio (NLR) to be a useful measure (Bhikram and Sandor, 2022). Several studies have proposed using inflammatory composite scores (Chat et al., 2021; Vinhaes et al., 2021), but when further explored using a factor analysis approach, these measures did not seem to perform better than individual proteins (Moriarity et al., 2021). While these approaches are useful, they do not reflect the activity/function of key immune proteins, unlike the novel multi-protein derived measure of IL-6 activity/bioavailability reported here.

Symptom-specificity in the context of inflammation-associated depression is well reported. Clinically, somatic symptoms (e.g., fatigue) rather than psychological symptoms (e.g., hopelessness) have been repeatedly implicated in patients with depression and evidence of inflammation (Kappelmann et al., 2021; Köhler-Forsberg et al., 2017; Milaneschi et al., 2021). We add to this evidence base, showing associations between a marker of IL-6 activity/bioavailability and somatic symptoms, fatigue, and depression severity. We also report associations between single inflammatory proteins and somatic symptoms (IL-6, CRP), fatigue (CRP), and depression severity (IL-6, CRP). This offers further reassurance that our novel measure of IL-6 activity/bioavailability is indeed a good representative of inflammation in this sample.

Regarding cognition, individual immune proteins have been linked to decreased psychomotor speed and reaction time (Bekhbat et al., 2021; Felger et al., 2016) and poorer performance in memory tasks (Dillon and Pizzagalli, 2018; Wachowska et al., 2022) in patients with depression. We report a negative association between our novel inflammatory biomarker and psychomotor speed, and with learning and memory before adjustment for confounders. Little evidence of an association was found between CRP and any of the cognitive outcomes assessed. However, IL-6 and sIL-6R were strongly associated with psychomotor speed, the latter being another novel finding of this study. Cognitive dysfunction is a core component of both DSM-V and ICD-10 diagnostic criteria for depression and currently available antidepressants do not seem to address such cognitive deficits (Capuron et al., 2002; Raison et al., 2006). This novel measure of IL-6 activity/bioavailability may provide further insight into the biological mechanisms contributing to cognitive dysfunction in depression, aiding future treatment development.

A key strength of the current study is our inclusion of a large battery of cognitive tasks designed to assess both hot and cold cognitive domains. This is critical as we work towards identifying domains most strongly associated with inflammation, for the purpose of inclusion in future research testing causality or therapeutic effect. We adjusted regression models for clinical and cognitive outcomes accordingly, considering several confounders known to be associated with inflammation, depression, and cognition, including age, sex, BMI, IQ, current antidepressant type, and treatment duration. Moreover, immune marker values in our patients with depression are likely to reflect low-grade inflammation as we systematically ruled out acute infection by several laboratory and clinical tests, including white blood cell count, antibody tests for TB, HIV, Hepatitis B, and Hepatitis C and a chest X-ray.

However, there are also limitations that must be considered. First, the sample size of this study is relatively small which limited the statistical power. Our sample also lacked diversity (>70% female and >90% white), thus reducing its potential generalisability. All participants had a diagnosis of depression and were taking anti-depressant medication and so, this sample may not be representative of all patients with depression. Moreover, while many cognitive tasks (including those presented here) have been well validated in case-control studies (e.g., comparing MDD and healthy individuals) where there may be large group differences, arguably these tests may be less sensitive at detecting individual differences (Hedge et al., 2018). The current investigation is also cross-sectional, and we must acknowledge the possibility that the above-presented associations between this novel biomarker and clinical and cognitive outcomes may differ over time. Nevertheless, this study adds value to our current knowledge base, presenting and testing a novel measure of inflammation for use in future immunopsychiatry investigations.

Further work is now needed to replicate our findings in larger, more diverse samples. Cell-based in vitro experiments will be helpful to assess the mechanistic validity of this ratio biomarker and its relationship to IL-6 trans-signalling. Longitudinal studies will be useful to examine whether this novel measure of IL-6 activity/bioavailability is associated with prognosis.

## 4 Conclusion

We report associations of a novel, multi-protein-derived biomarker of IL-6 activity/bioavailability with several clinical and cognitive outcomes in a sample of individuals with depression including somatic symptoms, fatigue, depression severity, and psychomotor speed. This novel biomarker performs well in comparison to individual inflammatory proteins often used in immunopsychiatry. Further research is needed to explore the potentially utility of this biomarker in other larger and more diverse samples, to establish the validity of this biomarker, and to assess its usefulness for disease risk and prognosis prediction.

## Supplementary Material

Supplementary data associated with this article can be found in the online version at doi:10.1016/j.psyneuen.2024.107008.

Supplementary Material

## Figures and Tables

**Table 1:** Characteristics of Insight Study participants with ICD-10 diagnosis of depression.

Characteristic	Total (N=86)
*Age*	
Mean (SD)	38.36 (11.79)
Median (Min, Max)	36.76 (20.84, 65.87)
*Sex, N* (%)	
Female	62 (72.09)
Male	24 (27.91)
*BMI* ^a^	
Mean (SD)	30.62 (8.80)
Median (Min, Max)	28.52 (17.43, 57.06)
*Ethnicity, N* (%)	
White	80 (93.02)
Other	6 (6.98)
*Educational Attainment, N* (%)	
No qualifications	1 (1.16)
GCSE/O levels	9 (10.47)
A levels	8 (9.30)
Vocational/College	16 (18.60)
University/Professional qualifications	52 (60.47)
*Employment Status, N* (%)^a^	
Unemployed	20 (23.53)
Employed	65 (76.47)
*Medication Type, N* (%)	
SSRI	63 (73.26)
Other	23 (26.74)
*Medication Duration (weeks)*	
Mean (SD)	97.40 (160.8)
Median (Min, Max)	52.00 (3.00, 1043)
*Depressive Episodes, N* (%)	
First episode	14 (16.28)
2–3	23 (26.74)
4–5	15 (17.44)
6 or more	34 (39.53)
*Estimated Premorbid IQ^b^*	
Mean (SD)	118.10 (5.87)
Median (Min, Max)	119.00 (101.30, 127.70)

ICD-10, International Classification of Diseases 10th Revision; SD, standard deviation; IQR, interquartile range; BMI, body mass index; SSRI, selective serotonin reuptake inhibitors.

^a^ N=85; ^b^N=84.

**Table 2:** Associations of IL-6 activity/bioavailability and other inflammatory markers with clinical and cognitive outcomes in depression.

Outcome		Exposure^a^	N^b^	Model 1	N^b^	Model 2	N^b^	Model 3		
				β (95% CI)		β (95% CI)		β (95% CI)	P	P_FDR_
*Clinical^c^*	*Somatic Symptoms*	IL-6 Activity/ Bioavailability	81	**1.09** **(0.37, 1.81)**	80	**1.03** **(0.23, 1.82)**	80	**1.09** **(0.30, 1.88)**	**0.01**	**0.01**
		IL-6	82	**0.92** **(0.18, 1.66)**	81	**1.78** **(0.78, 2.79)**	81	**1.92** **(0.87, 2.97)**	**4.94e-04**	**1.71e-03**
		CRP	86	**1.44** **(0.75, 2.13)**	85	**1.60** **(0.76, 2.45)**	85	**1.48** **(0.63, 2.33)**	**8.57e-04**	**1.71e-03**
		sIL-6R	82	0.73 (−0.02, 1.48)	81	0.57 (−0.24, 1.37)	81	0.63 (−0.21, 1.48)	0.14	0.14
	*Fatigue*	IL-6 Activity/ Bioavailability	81	**4.75** **(1.88, 7.61)**	80	**3.65** **(0.53, 6.78)**	80	**4.34** **(1.26, 7.42)**	**0.01**	**0.03**
		IL-6	82	**4.57** **(1.70, 7.44)**	81	3.29 (−0.82, 7.39)	81	4.06 (−0.20, 8.32)	0.06	0.08
		CRP	86	**5.37** **(2.69, 8.04)**	85	**4.18** **(0.90, 7.45)**	85	**3.80** **(0.53, 7.07)**	**0.02**	**0.047**
		sIL-6R	82	2.40 (−0.60, 5.40)	81	1.01 (−2.12, 4.14)	81	1.80 (−1.45, 5.05)	0.27	0.27
	*Depression Severity*	IL-6 Activity/ Bioavailability	81	**2.60** **(0.52, 4.69)**	80	**2.64** **(0.30, 4.98)**	80	**3.06** **(0.71, 5.40)**	**0.01**	**0.02**
		IL-6	82	**2.36** **(0.23, 4.49)**	81	**4.06** **(1.03, 7.09)**	81	**4.80** **(1.62, 7.97)**	**3.58e-03**	**0.01**
		CRP	86	**3.15** **(1.12, 5.18)**	85	**3.54** **(1.03, 6.05)**	85	**3.33** **(0.80, 5.86)**	**0.01**	**0.02**
		sIL-6R	82	1.55 (−0.62, 3.72)	81	1.30 (−1.07, 3.66)	81	2.02 (−0.46, 4.51)	0.11	0.11
	*Quality of life*	IL-6 Activity/ Bioavailability	81	**−0.07** **(−0.13, −0.01)**	80	− 0.06 (−0.12, 0.01)	80	**−0.07** **(−0.13, −0.01)**	**0.03**	**0.045**
		IL-6	82	**−0.08** **(−0.14, −0.02)**	81	**-0.12** **(−0.20, −0.04)**	81	**-0.12** **(−0.21, −0.04)**	**4.01e-03**	**0.01**
		CRP	86	**−0.10** **(−0.16, −0.05)**	85	**−0.11** **(−0.17, −0.04)**	85	**−0.09** **(−0.16, −0.03)**	**0.01**	**0.01**
		sIL-6R	82	− 0.04 (−0.10, 0.02)	81	−0.03 (−0.09, 0.04)	81	− 0.05 (−0.11, 0.02)	0.16	0.16
*Cognitive^d^*	*Reaction Time*	IL-6 Activity/ Bioavailability	77	4.98 (−6.63, 16.58)	77	6.15 (−5.81, 18.11)	77	5.88 (−6.13, 17.89)	0.33	0.51
		IL-6	77	6.70 (−4.71, 18.11)	77	6.76 (−4.70, 18.22)	77	3.89 (−7.72, 15.49)	0.51	0.51
		CRP	80	6.97 (−3.84, 17.79)	80	7.18 (−3.71, 18.07)	80	5.89 (−5.30, 17.09)	0.30	0.51
		sIL-6R	77	3.15 (−8.68, 14.98)	77	4.02 (−8.08, 16.12)	77	4.80 (−7.64, 17.23)	0.44	0.51
	*Psychomotor Speed*	IL-6 Activity/ Bioavailability	78	**−5.59** **(−9.34, −1.84)**	78	**−5.49** **(−9.29, −1.69)**	78	**−5.46** **(−9.09, −1.84)**	**3.67e-03**	**0.01**
		IL-6	78	**−5.71** **(−9.42, −2.00)**	78	**−5.80** **(−9.52, −2.08)**	78	**−5.12** **(−8.72, −1.53)**	**0.01**	**0.01**
		CRP	81	− 2.72 (−6.48, 1.05)	81	− 2.67 (−6.45, 1.12)	81	− 2.48 (−6.19, 1.24)	0.19	0.19
		sIL-6R	78	**−6.10** **(−9.85, −2.35)**	78	**−6.01** **(−9.79, −2.22)**	78	**−6.03** **(−9.76, −2.30)**	**1.90e-03**	**0.01**
	*Learning and Memory*	IL-6 Activity/ Bioavailability	77	**−2.72** **(−5.35, −0.10)**	77	− 2.17 (−4.84, 0.49)	77	−1.45 (−4.04, 1.15)	0.27	0.81
		IL-6	77	0.23 (−2.44, 2.90)	77	0.27 (−2.33, 2.88)	77	0.83 (−1.68, 3.34)	0.51	0.81
		CRP	80	0.38 (−2.15, 2.91)	80	0.55 (−1.94, 3.04)	80	0.46 (−1.97, 2.88)	0.71	0.81
		sIL-6R	77	−1.97 (−4.68, 0.73)	77	−1.46 (−4.17, 1.25)	77	− 0.33 (−3.03, 2.37)	0.81	0.81

BMI, body mass index; CRP, C-reactive protein; FDR, false discovery rate; IL-6, interleukin 6; NART, National Adult Reading Test; sIL-6R, soluble IL-6 receptor.

a IL-6, CRP, and sIL-6R violated the assumption of normality and thus were log-transformed before analysis.

b N per analysis differs due to missing data. All analyses were performed using maximum data available.

c Regression models including clinical outcomes were adjusted as follows: Model 1 = unadjusted, Model 2 = adjusted for BMI, Model 3 = additionally adjusted for age, sex, current anti-depressant type, treatment duration, and number of depressive episodes.

d Regression models including cognitive outcomes were adjusted as follows: Model 1 = unadjusted, Model 2 = adjusted for NART score, Model 3 = additionally adjusted for age, sex, current anti-depressant type, treatment duration, and number of depressive episodes.
