# Supplementary material for: A novel biomarker of interleukin 6 activity and clinical and cognitive outcomes in depression
Source: Psychoneuroendocrinology. Author manuscript; Available in PMC 2025 May 27. (PMC7617704; doi:10.1016/j.psyneuen.2024.107008)

**Supplementary Material**

**Foley *et al*. A novel biomarker of interleukin 6 activity and clinical and cognitive outcomes in depression**

Table of Contents

[1. Supplementary Abbreviations 2](#_Toc156906666)

[2. Inclusion and Exclusion Criteria for The Insight Study 3](#_Toc156906667)

[3. Supplementary Tables 4](#_Toc156906668)

[Supplementary Table 1. 4](#_Toc156906669)

[Supplementary Table 2 5](#_Toc156906670)

[Supplementary Table 3 6](#_Toc156906671)

[Supplementary Table 4. 7](#_Toc156906672)

[Supplementary Table 5 8](#_Toc156906673)

[Supplementary Table 6 12](#_Toc156906674)

[Supplementary Table 7. 13](#_Toc156906675)

[4. Supplementary Figures 14](#_Toc156906676)

[Supplementary Figure 1. 14](#_Toc156906677)

[Supplementary Figure 2. 15](#_Toc156906678)

[Supplementary Figure 3. 16](#_Toc156906679)

# **Supplementary Abbreviations**

- BDI = Beck’s Depression Inventory
- BMI = body mass index
- CIS-R = Clinical Interview Schedule – Revised
- CRP = C-reactive protein
- FDR = False discovery rate
- ICD-10 = International Classification of Diseases-10
- IFNy = Interferon gamma
- IL = Interleukin
- IQR = Interquartile range
- NART = National Adult Reading Test
- SD = Standard deviation
- Sgp130 = Soluble gp130
- SIL-6R = Soluble IL-6 receptor
- TNF⍺ = Tumour necrosis factor alpha

# **Inclusion and Exclusion Criteria for The Insight Study**

All participants were required to meet the following criteria, assessed via self-report and verified by the participant’s general practitioner prior to enrolment: aged 20-65 years, ICD-10 criteria for depressive episode at time of assessment (confirmed by the CIS-R), currently taking an antidepressant at an adequate dose (as determined by British National Formulary) for at least four weeks. Exclusion criteria were current or lifetime diagnosis of bipolar disorder, psychotic disorder, personality disorder, eating disorder, history of alcohol or substance abuse/dependence within six months prior to assessment (nicotine and caffeine dependence were not exclusionary), current suicidal thoughts or wishes (assessed by BDI-II item 9 score of 3) or history of suicide attempt or deliberate self-harm within six months prior to assessment, any current infection, any infection requiring hospitalisation or treatment with intravenous antibiotics within four weeks prior to assessment, pregnancy or breast feeding, and physical illness and/or use of medication likely to compromise interpretation of immunological data. Further exclusionary criteria were applied to participants recruited between March 2021 and May 2022, due to known increased risk of COVID-19 contraindications. These included lifetime history of respiratory illness (e.g., asthma), non-white ethnicity, and a BMI >35.

# **Supplementary Tables**

Supplementary Table 1. Lower and upper detection limits for included inflammatory proteins.

|  | **Lower limit of detection (LLOD)** | **Upper limit of detection (ULOD)** | **Number of participants below LLOD** |
| --- | --- | --- | --- |
| **High sensitivity CRP** (mg/L) | 0.2 | ~18* | 4 |
| **IFNᵧ**  (pg/mL) | 1.9 | 3100 | 0 |
| **IL-2**  (pg/mL) | 0.5 | 2840 | 77 |
| **IL-4**  (pg/mL) | 0.1 | 512 | 73 |
| **IL-6**  (pg/mL) | 0.5 | 1456 | 23 |
| **Soluble IL-6 receptor** (ng/mL) | 0.03 | 100 | 0 |
| **IL-8**  (pg/mL) | 0.3 | 1142 | 0 |
| **IL-10**  (pg/mL) | 0.2 | 748 | 28 |
| **IL-12p70**  (pg/mL) | 0.3 | 986 | 52 |
| **IL-13**  (pg/mL) | 1.3 | 992 | 53 |
| **Soluble gp130**  (ng/mL) | 8 | 1600 | 0 |
| **TNF⍺**  (pg/mL) | 0.3 | 784 | 0 |

*samples repeated on dilution

**Supplementary Table 2. Soluble gp130 batch effect exploration.** Sgp130 was assayed separately for high (batch 1) and low (batch 2) CRP groups. Descriptive table of sgp130 for all participants, batch 1, and batch 2 are presented to explore the presence of a possible batch effect. There was no evidence of large batch effect.

| **Participants** | **N** | **Mean (SD)** | **Median (IQR)** | **Minimum** | **Maximum** |
| --- | --- | --- | --- | --- | --- |
| **All** | 81 | 277.56  (47.63) | 284.40  (240.00 - 319.00) | 161.80 | 358.10 |
| **Batch 1** | 28 | 232.04  (37.73) | 232.25  (213.35 - 255.07) | 161.80 | 320.20 |
| **Batch 2** | 53 | 301.62  (32.33) | 303.20  (282.90 - 326.60) | 222.40 | 358.10 |

**Supplementary Table 3.** Primary and secondary outcome measures.

|  | **Name of Scale/Test** | **Outcome measure** | **Outcome type** |
| --- | --- | --- | --- |
| **Clinical** | Beck’s Depression Inventory II –Somatic symptom score | Somatic symptoms | Primary |
|  | Multidimensional Fatigue Inventory | Fatigue |  |
|  | Beck’s Depression Inventory II | Depression severity | Secondary |
|  | Snaith-Hamilton Pleasure Scale | Anhedonia |  |
|  | State-Trait Anxiety Inventory – State | State anxiety |  |
|  | State-Trait Anxiety Inventory – Trait | Trait anxiety |  |
|  | EQ-5D three-level version | Quality of life |  |
| **Cognitive** | Digit symbol coding test | Psychomotor speed | Primary |
|  | CANTAB Reaction Time Test five-choice mode | Reaction time |  |
|  | CANTAB Paired Associates Learning test | Visual associative learning and memory | Secondary |
|  | CANTAB One Touch Stockings of Cambridge test | Executive function |  |
|  | CANTAB Rapid Visual Information Processing test | Sustained attention |  |
|  | Emotional Categorisation and Recall Task | Affective bias |  |
|  | CANTAB Emotion Bias Task | Emotional perceptual bias |  |

**Supplementary Table 4.** Inflammatory protein descriptives table.

| **Immune Protein** | **Mean (SD)** | **Median (IQR)** |
| --- | --- | --- |
| **High sensitivity CRP**  (mg/L) | 4.50  (5.06) | 2.85  (0.20 – 25.05) |
| **IFNᵧ**  (pg/mL) | 7.44  (4.94) | 5.90  (2.52 – 27.07) |
| **IL-2**  (pg/mL) | 0.51  (0.10) | 0.50  (0.50 – 1.33) |
| **IL-4**  (pg/mL) | 0.11  (0.02) | 0.10  (0.10 – 0.22) |
| **IL-6**  (pg/mL) | 1.09  (0.84) | 0.81  (0.50 – 5.59) |
| **Soluble IL-6 receptor**  (ng/mL) | 45.60  (12.47) | 43.97  (27.05 – 87.74) |
| **IL-8**  (pg/mL) | 11.84  (5.02) | 10.77  (4.10 – 33.43) |
| **IL-10**  (pg/mL) | 0.31  (0.17) | 0.22  (0.20 – 1.11) |
| **IL-12p70**  (pg/mL) | 0.42  (0.38) | 0.30  (0.30 – 2.78) |
| **IL-13**  (pg/mL) | 1.61  (0.91) | 1.30  (1.30 – 7.21) |
| **Soluble gp130**  (ng/mL) | 277.56  (47.63) | 284.40  (161.80 – 358.10) |
| **TNF⍺**  (pg/mL) | 1.58  (0.56) | 1.52  (0.81 – 5.15) |

**Supplementary Table 5. Associations of IL-6 activity/bioavailability and other inflammatory markers with remaining clinical and cognitive outcomes in depression.**

| **Outcome** | | **Exposure^a^** | **N^b^** | **Model 1** | **N^b^** | **Model 2** | **N^b^** | **Model 3** | | |
| --- | --- | --- | --- | --- | --- | --- | --- | --- | --- | --- |
|  |  |  |  | **β (95% CI)** |  | **β (95% CI)** |  | **β (95% CI)** | **P** | **P_FDR_** |
| *Clinical^c^* | *Anhedonia* | IL-6 Activity/  Bioavailability | 81 | 0.07  (-0.73, 0.86) | 80 | -0.06  (-0.92, 0.80) | 80 | 0.11  (-0.72, 0.94) | 0.79 | 0.99 |
|  |  | IL-6 | 82 | 0.55  (-0.23, 1.34) | 81 | -0.10  (-1.22, 1.01) | 81 | 0.03  (-1.09, 1.15) | 0.96 | 0.99 |
|  |  | CRP | 86 | 0.34  (-0.42, 1.10) | 85 | 0.14  (-0.78, 1.06) | 85 | 0.01  (-0.92, 0.93) | 0.99 | 0.99 |
|  |  | sIL-6R | 82 | -0.12  (-0.92, 0.67) | 81 | -0.26  (-1.10, 0.58) | 81 | -0.06  (-0.90, 0.78) | 0.88 | 0.99 |
|  | *State Anxiety* | IL-6 Activity/  Bioavailability | 81 | 0.35  (-2.00, 2.70) | 80 | 0.28  (-2.36, 2.92) | 80 | 0.49  (-2.22, 3.21) | 0.72 | 0.72 |
|  |  | IL-6 | 82 | 1.92  (-0.38, 4.22) | 81 | 3.17  (-0.14, 6.48) | 81 | **3.63**  **(0.09, 7.18)** | **0.04** | 0.18 |
|  |  | CRP | 86 | 1.53  (-0.68, 3.74) | 85 | 1.96  (-0.78, 4.69) | 85 | 1.72  (-1.11, 4.54) | 0.23 | 0.46 |
|  |  | sIL-6R | 82 | -0.88  (-3.21, 1.45) | 81 | -1.16  (-3.70, 1.37) | 81 | -1.10  (-3.82, 1.63) | 0.43 | 0.57 |
|  | *Trait Anxiety* | IL-6 Activity/  Bioavailability | 81 | 0.56  (-1.38, 2.50) | 80 | 0.75  (-1.43, 2.92) | 80 | 1.08  (-1.13, 3.30) | 0.33 | 0.79 |
|  |  | IL-6 | 82 | 0.27  (-1.67, 2.20) | 81 | 0.63  (-2.17, 3.44) | 81 | 0.72  (-2.26, 3.70) | 0.63 | 0.79 |
|  |  | CRP | 86 | -0.20  (-2.06, 1.66) | 85 | -0.32  (-2.63, 1.98) | 85 | -0.32  (-2.65, 2.02) | 0.79 | 0.79 |
|  |  | sIL-6R | 82 | 0.05  (-1.88, 1.99) | 81 | 0.08  (-2.03, 2.20) | 81 | 0.66  (-1.57, 2.90) | 0.56 | 0.79 |
| *Cognitive^d^* | *Emotional perceptual bias* | IL-6 Activity/  Bioavailability | 75 | 0.20  (-0.13, 0.54) | 75 | 0.25  (-0.09, 0.60) | 75 | 0.24  (-0.10, 0.57) | 0.16 | 0.66 |
|  |  | IL-6 | 75 | 0.05  (-0.26, 0.37) | 75 | 0.05  (-0.26, 0.37) | 75 | -0.02  (-0.34, 0.29) | 0.88 | 0.88 |
|  |  | CRP | 78 | 0.06  (-0.25, 0.37) | 78 | 0.07  (-0.24, 0.38) | 78 | 0.05  (-0.26, 0.36) | 0.75 | 0.88 |
|  |  | sIL-6R | 75 | 0.16  (-0.17, 0.50) | 75 | 0.20  (-0.14, 0.54) | 75 | 0.15  (-0.19, 0.49) | 0.37 | 0.75 |
|  | *Reaction Time: Positive Valence* | IL-6 Activity/  Bioavailability | 70 | -61.79  (-138.18, 14.59) | 70 | -58.00  (-137.14, 21.13) | 70 | -52.48  (-135.06, 30.09) | 0.21 | 0.42 |
|  |  | IL-6 | 70 | -27.08  (-101.09, 49.93) | 70 | -26.62  (-100.90, 47.66) | 70 | -30.01  (-106.72, 46.69) | 0.44 | 0.49 |
|  |  | CRP | 73 | -64.59  (-135.57, 6.38) | 73 | -61.66  (-132.69, 9.36) | 73 | -62.60  (-136.06, 10.86) | 0.09 | 0.37 |
|  |  | sIL-6R | 70 | -33.98  (-110.96, 42.99) | 70 | -29.57  (-108.24, 49.10) | 70 | -28.67  (-112.10, 54.77) | 0.49 | 0.49 |
|  | *Reaction Time: Negative Valence* | IL-6 Activity/  Bioavailability | 70 | -61.27  (-136.45, 13.90) | 70 | -68.19  (-145.84, 9.47) | 70 | -68.93  (-149.12, 11.26) | 0.09 | 0.28 |
|  |  | IL-6 | 70 | -18.11  (-91.12, 54.90) | 70 | -18.33  (-91.84, 55.19) | 70 | -19.94  (-95.41, 55.53) | 0.60 | 0.60 |
|  |  | CRP | 73 | -54.92  (-123.51, 13.66) | 73 | -55.12  (-124.38, 14.15) | 73 | -53.96  (-126.45, 18.52) | 0.14 | 0.28 |
|  |  | sIL-6R | 70 | -37.63  (-113.29, 38.03) | 70 | -41.37  (-118.75, 36.02) | 70 | -51.62  (-132.75, 29.52) | 0.21 | 0.28 |
|  | *Total Positive Recall* | IL-6 Activity/  Bioavailability | 81 | -0.19  (-0.69, 0.32) | 81 | -0.16  (-0.67, 0.35) | 81 | -0.16  (-0.67, 0.34) | 0.52 | 0.98 |
|  |  | IL-6 | 81 | -0.18  (-0.68, 0.33) | 81 | -0.19  (-0.69, 0.32) | 81 | -0.09  (-0.60, 0.41) | 0.71 | 0.98 |
|  |  | CRP | 84 | -0.11  (-0.60, 0.38) | 84 | -0.09  (-0.59, 0.40) | 84 | 0.003  (-0.50, 0.50) | 0.99 | 0.99 |
|  |  | sIL-6R | 81 | -0.12  (-0.63, 0.39) | 81 | -0.10  (-0.62, 0.41) | 81 | 0.09  (-0.61, 0.43) | 0.74 | 0.98 |
|  | *Total Negative Recall* | IL-6 Activity/  Bioavailability | 81 | 0.17  (-0.28, 0.62) | 81 | 0.18  (-0.27, 0.64) | 81 | 0.26  (-0.19, 0.72) | 0.25 | 0.50 |
|  |  | IL-6 | 81 | -0.22  (-0.66, 0.23) | 81 | -0.63  (-0.67, 0.23) | 81 | -0.101  (-0.56, 0.36) | 0.66 | 0.75 |
|  |  | CRP | 84 | -0.23  (-0.67, 0.21) | 84 | -0.22  (-0.66, 0.23) | 84 | -0.07  (-0.53, 0.38) | 0.75 | 0.75 |
|  |  | sIL-6R | 81 | 0.29  (-0.16, 0.74) | 81 | 0.30  (-0.16, 0.75) | 81 | 0.38  (-0.08, 0.858) | 0.11 | 0.43 |
|  | *Executive Function* | IL-6 Activity/  Bioavailability | 75 | -0.23  (-0.83, 0.36) | 75 | -0.14  (-0.75, 0.47) | 75 | -0.19  (-0.81, 0.43) | 0.54 | 0.80 |
|  |  | IL-6 | 75 | 0.05  (-0.51, 0.61) | 75 | 0.06  (-0.50, 0.61) | 75 | 0.07  (-0.50, 65) | 0.80 | 0.80 |
|  |  | CRP | 78 | 0.09  (-0.45, 0.63) | 78 | 0.11  (-0.43, 0.65) | 78 | 0.10  (-0.46, 0.66) | 0.72 | 0.80 |
|  |  | sIL-6R | 75 | -0.12  (-0.71, 0.47) | 75 | -0.04  (-0.64, 0.55) | 75 | -0.08  (-0.71, 0.54) | 0.79 | 0.80 |
|  | *Sustained Attention* | IL-6 Activity/  Bioavailability | 77 | -1.18  (-19.74, 17.38) | 77 | -0.69  (-19.89, 18.52) | 77 | -3.68  (-22.09, 14.73) | 0.69 | 0.88 |
|  |  | IL-6 | 77 | 8.51  (-9.71, 26.73) | 77 | 8.55  (-9.79, 26.88) | 77 | 2.14  (-15.60, 19.88) | 0.81 | 0.88 |
|  |  | CRP | 80 | -5.40  (-23.32, 12.51) | 80 | -5.13  (-23.19, 12.92) | 80 | -10.65  (-27.62, 6.32) | 0.21 | 0.86 |
|  |  | sIL-6R | 77 | 3.40  (-15.44, 22.25) | 77 | 4.00  (-15.33, 23.33) | 77 | 1.39  (-17.64, 20.42) | 0.88 | 0.88 |

^a^IL-6, CRP, and sIL-6R violated the assumption of normality and thus were log-transformed before analysis.

^b^N per analysis differs due to missing data. All analyses were performed using maximum data available.

^c^Regression models including clinical outcomes were adjusted as follows: Model 1 = unadjusted, Model 2 = adjusted for BMI, Model 3 = additionally adjusted for age, sex, current anti-depressant type, and treatment duration.

^d^Regression models including cognitive outcomes were adjusted as follows: Model 1 = unadjusted, Model 2 = adjusted for NART score, Model 3 = additionally adjusted for age, sex, current anti-depressant type, and treatment duration.

**Supplementary Table 6. Dissociation constant sensitivity analyses.** Sensitivity analyses were performed to test the effect of substituting the dissociation constants in the IL-6 bioavailability/activity marker calculation with values 10 times higher and 10 times lower. The re-calculated ratio scores were used in an unadjusted linear regression analysis with total depression severity score and the estimates and confidence intervals compared for each condition.

| **Dissociation Constants** | **N** | **Beta (95% CI)** | **P** | **FDR-adjusted P** |
| --- | --- | --- | --- | --- |
| Original values | 81 | 73.25  (14.55-131.95) | 0.015 | 0.022 |
| Decreased values | 81 | 1006.98  (279.5-1734.47) | 0.007 | 0.022 |
| Increased values | 81 | 14.49  (2.13-26.84) | 0.022 | 0.022 |

**Supplementary Table 7.** Sensitivity analyses assessing whether the ratio score was primarily driven by an individual inflammatory marker used in its creation (i.e., IL-6, sIL-6R, sgp130). The new ratio scores were used as the exposure in our unadjusted multivariable linear regression analyses with depression severity as the outcome.

| **Ratio Measure** | **N** | **Beta (95% CI)** | **P** |
| --- | --- | --- | --- |
| **Ratio** | 81 | 2.60 (0.52-4.69) | **0.02** |
| **Ratio (IL-6 x3)** | 81 | 2.61 (0.52-4.69) | **0.02** |
| **Ratio (sIL-6R x3)** | 81 | 2.67 (0.59-4.75) | **0.01** |
| **Ratio (sgp130 x3)** | 81 | 2.24 (0.13-4.35) | **0.04** |

# **Supplementary Figures**

**Supplementary Figure 1. Sgp130 batch effect exploration.** Sgp130 was assayed separately for high (batch 1) and low (batch 2) CRP groups. Distributions of (**A**) batch 1 vs (**B**) batch 2 are presented. No evidence of a large batch effect was found.

**A**

**
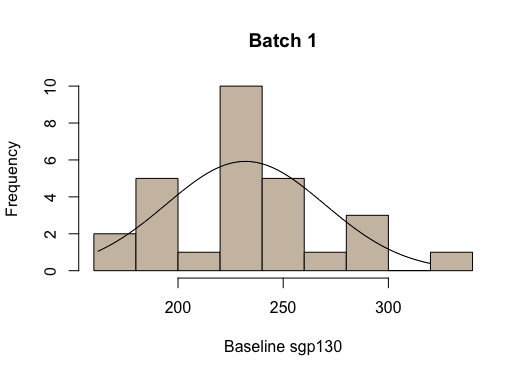
**

sgp130 levels

**B**


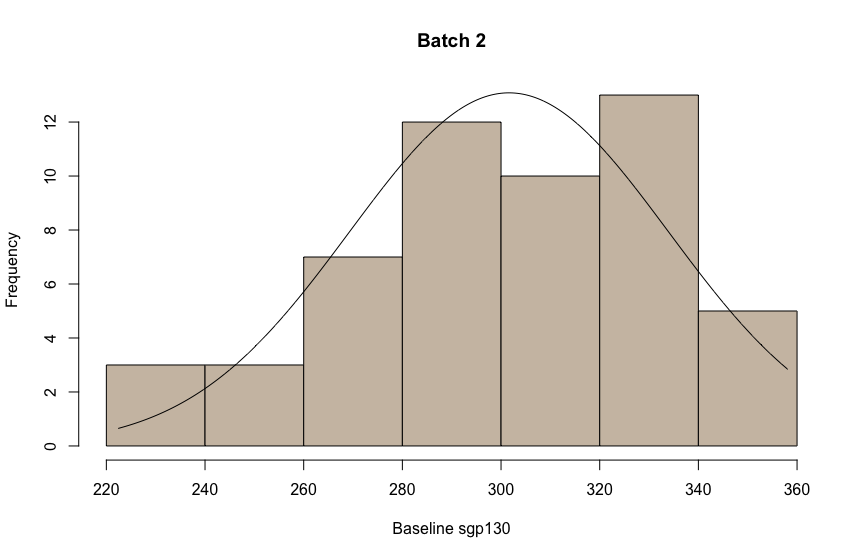


sgp130 levels

**Supplementary Figure 2. Correlation of novel IL-6 activity/bioavailability biomarker with other inflammatory markers.** Strength of correlation is represented as a colour gradient and numerically for each pair of markers.


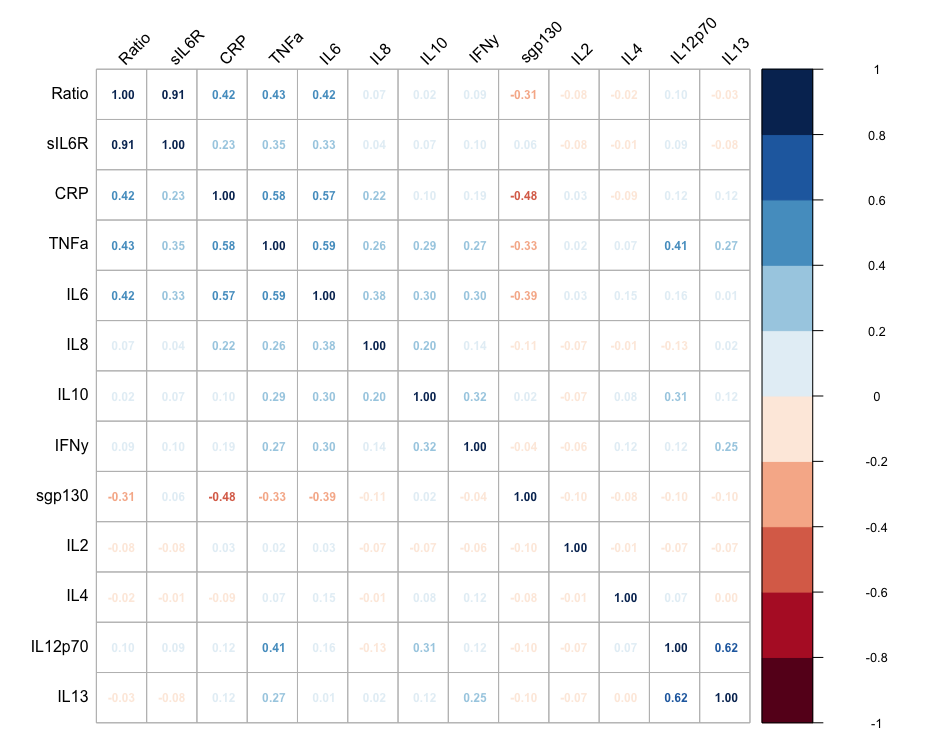


**Supplementary Figure 3. Binding affinity sensitivity analyses.** Binding affinity was estimated when calculating the novel IL-6 bioavailability/activity marker and represented by dissociation constants. Sensitivity analyses were performed on total depression severity score to compare dissociation constant values in three conditions: **(A)** 10 times lower, **(B)** original values, and **(C)** 10 times higher.

**A**

**
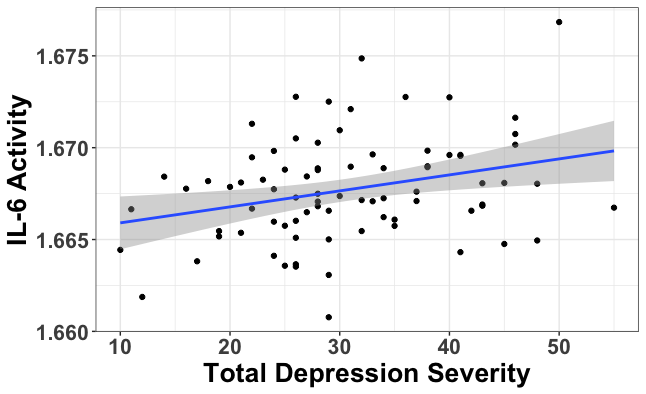
**

**B**

**
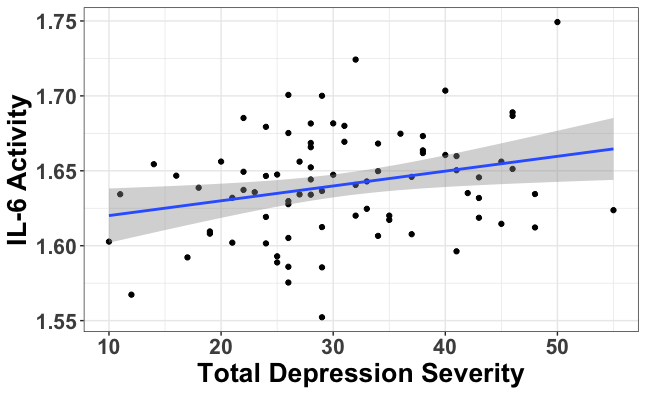
**

**C**


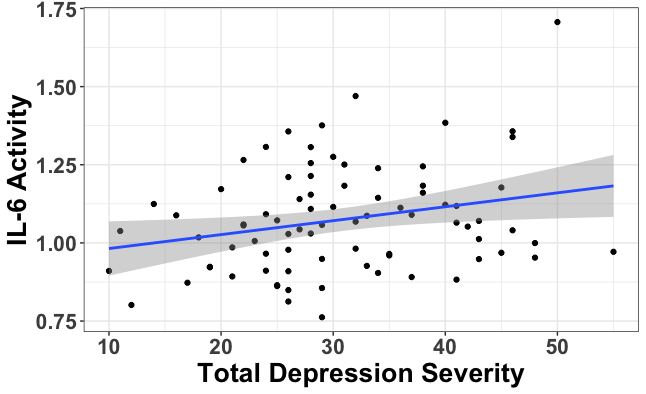

Supplement: Supplementary Material [file EMS205671-supplement-Supplementary_Material.docx]
